# Recent Advances in Basalt Fiber Reinforced Asphalt Mixture for Pavement Applications

**DOI:** 10.3390/ma15196826

**Published:** 2022-10-01

**Authors:** Yingxin Hui, Guangyu Men, Peng Xiao, Qin Tang, Fangyuan Han, Aihong Kang, Zhengguang Wu

**Affiliations:** 1 Ningxia Communications Construction Co., Ltd., Yinchuan 750004, China; 2 Ningxia Road Maintenance Engineering Technology Research Center, Yinchuan 750004, China; 3 College of Architectural Science and Engineering, Yangzhou University, Yangzhou 225127, China; 4 Research Center for Basalt Fiber Composite Construction Materials, Yangzhou University, Yangzhou 225127, China

**Keywords:** basalt fiber, fiber-reinforced asphalt mixture, asphalt pavement performance, fiber surface modifier

## Abstract

This paper conducts a thorough review of the literature on the feasibility and current state-of-the-art incorporation of basalt fiber (BF) into asphalt pavement materials, focusing on fiber characteristics, dosage, incorporation methods, mixture properties, and surface modification techniques. The optimum basalt fiber dosage should be determined based on engineering performance parameters such as asphalt type, fatigue cracking, thermal cracking, rutting, and moisture resistance of asphalt mixtures. Basalt fibers are added to asphalt mixes by dry method or mixed method to achieve better dispersion. Adding BF to asphalt mixtures increased performance characteristics like cracking resistance, rutting resistance, and fatigue resistance. Overall, incorporating BF into asphalt mixtures would lower costs while increasing pavement service life. More research is needed to fully understand the effects of different sizes of BF on pavement performance and the possible environmental and economic repercussions of fiber surface alteration.

## 1. Introduction

The demand for road building has been increasing due to the rapid rise of urbanization and traffic volume worldwide. Road pavement materials performance needs to improve, with road stability and long-term durability being the primary concerns [1]. Asphalt pavement is more flexible than concrete pavement, with low noise, good skid resistance, good driving comfort, high stability, extended functional plasticity, and other properties that allow it to be used in broader applications [2]. Fiber-reinforced asphalt, a composite material, was chosen as the pavement material, and it was demonstrated that the fiber could greatly increase resistance to the flow, rutting, water damage, and fatigue cracking of asphalt [3,4].

This article focuses on basalt fiber (BF), an ecofriendly fiber material. BF is a high-performance inorganic fiber made from natural basalt that is recognized as a green industrial material in the 21st century. Basalt is well-known for its safety and plenty of reserves, as well as its thermal stability, strength, and durability [5]. BFs are lighter and have equivalent mechanical qualities to steel fibers. Basalt fibers can be regarded as environmentally lower damaging reinforcements since they consume less energy and emit less CO_2_ [6]. The production method is similar to that of glass fibers, but it consumes less energy, includes no additives, and is less expensive than glass and carbon fibers, offering an extra economic benefit [7]. Overall, BF is utilized as a reinforcing material in various areas because it is cost-effective and environmentally friendly, with the added benefits of acid and alkali resistance, high strength, and resilience to both high and low temperatures [8,9,10].

Today, increased traffic volume causes increased difficulty in pavement loading conditions, extreme environmental conditions cause increased road stability requirements, and basalt fiber asphalt mixes are considered an effective material for improving road performance such as anti-fatigue, anti-water damage, and anti-cracking [11,12]. Currently, scholars studying the exploration and application of fiber-reinforced asphalt mixtures range from improving road performance to finding alternative materials with low energy consumption and high performance returns, from the study of the effect of fiber size or doping on the simple parameters of the mixture to the detailed analysis of the physical and chemical parameters of various aspects of fiber materials and the suitability of asphalt mixtures [13,14]. To explain the impact of BF reinforcement, several researchers proposed a Viscosity-enhancing and stability mechanism and a Reinforcement and bridging mechanism [15]. BFs added to hot-mix asphalt mixes were shown to enhance the service life of road and airport runway pavements by up to 1.5 times [16]. It can be observed that BF research is progressively gaining traction. Since the study and use of BF reinforced asphalt materials are still in its early stages, most prior studies have concentrated on a single performance improvement, the influence of a single fiber parameter, or the application of a road portion of the effect research [17]. It is difficult to guide the selection, design, and construction applications of BF, or to conduct a comprehensive road performance evaluation research.

This work aims to offer an overview of the physical characteristics and chemical composition of BFs used in asphalt pavements and the methods used to prepare BF asphalt mixture, the road performance of BF asphalt pavements, and the BF surface modifiers. Based on this, current research findings and recommendations are presented in each area to provide a rich reference for the study of BF asphalt mixes, an ecologically friendly pavement material, and the design and development of road pavement materials.

## 2. Basalt Fiber Materials

### 2.1. Properties of Basalt Fiber

#### 2.1.1. Chemical Properties of Basalt Fiber

Basalt is a black, fine-grained, solidified volcanic rock that is hard, thick, and inert. Basalt is mainly composed of SiO_2_, Al_2_O_3_, Fe_2_O_3_, FeO, CaO, and MgO. Basalt is classified into alkaline basalt (up to 42% SiO_2_), slightly acidic basalt (43–46% SiO_2_), and acidic basalt (over 46% SiO_2_). Only acidic basalts can fulfill the fiber processing criteria [5]. The thermal and chemical stability and mechanical and physical qualities of monofilament fibers are affected by the elemental composition, chemical composition, mineral composition, and production procedure of basalt [8]. Microstructure and elemental composition studies of BF, for example, demonstrate that Al_2_O_3_ in BFs improves the uniform distribution of flaws and plays a vital role in the material composition in favor of the mechanical characteristics of the fibers. The redox state of iron affected Fe_2_O_3_ in BF, and high Fe^3+^ concentration in total iron promoted spontaneous crystallization and increased conversion of Fe^2+^ to Fe^3+^ in the material, increasing the number of defects on the fiber surface while lowering tensile strength [18]. Mineral composition in basalt affects the tensile strength of BF [19].

#### 2.1.2. Physical Properties of Basalt Fiber

The most important physical qualities to consider when analyzing fibers are tensile strength, modulus of elasticity, elongation at break, and heat resistance temperature. These properties have a direct impact on the binder and mixture performance. The essential features of BFs utilized in asphalt mixes are summarized in Table 1. BFs have a density of 2.46–3.3 g/cm^3^, a tensile strength of 1100–4850 MPa, a modulus of elasticity of 40–110 GPa, a break elongation of 2.5–3.3%, and a heat resistance of 170–180 °C. The BF possesses properties such as high temperature-resistance, high tensile strength, high corrosion resistance, and UV radiation, and eco-friendly [10].

#### 2.1.3. Production Process of Basalt Fiber

**Figure 1:**
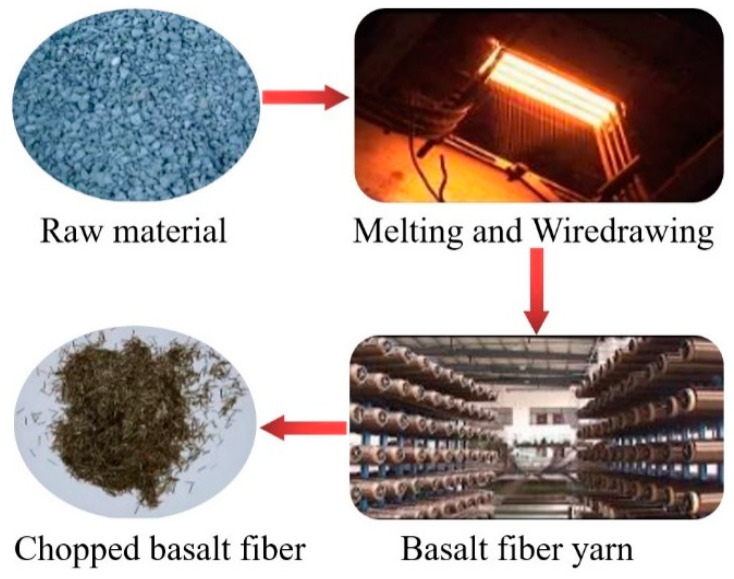
Preparation process of basalt fiber.

The following activities are involved in the production of continuous BFs: raw material preparation; rock melting; melt homogeneity and supply to bushings; melt drawing via bushing units; drawing of elementary filaments, application of sizing agent, and winding on bobbins. Figure 1 depicts the process of producing basalt fiber. The furnace chamber temperature, fiber stretching temperature, and fiber size all significantly impact the ultimate mechanical characteristics of the BF material during the manufacturing process [5]. The sizing stage in the preparation process functions as “clothing” for the fibers, substantially lowering the stress concentration of surface flaws by blunting the crack tip, boosting the strength of BFs by more than 15% [18].

### 2.2. Adsorption of Basalt Fibers to Asphalt

Fibers may adsorb a certain amount of asphalt in an asphalt mixture, and the rate of oil adsorption can impact the performance of the asphalt mixture. The greater the adsorption rate of a fiber-reinforced asphalt mixture within a specific range, the less likely it is that oil leaks, rutting, and other pavement degradation will occur at high temperatures. As a result, the capacity of fibers to adsorb asphalt is a critical signal in assessing the performance of asphalt mastics or mixtures. Kou et al. [50] utilized the basket leak method; the oil adsorption rate of lignin fiber is 8.4 times its weight, while the BF oil adsorption rate is only half that of the lignin fiber. Since the specific surface area of the lignin fibers is 12.87 times that of the BFs, it is assumed that they have a more extensive contact area with the asphalt and a higher oil adsorption rate. Microscopic photos also demonstrated that lignin fibers intertwine and build mechanically riveting structures to carry more asphalt, contributing to the increased oil adsorption of lignin fiber. Xing et al. [51] found that the adsorption rate of lignin fibers was 852.4%, and that of BFs was 272.4% based on the Brinell funnel technique. This is due to the BF density, smoothness, and low adsorption rate to asphalt. The fiber adsorption capacity of asphalt, to some extent, reflects the stabilizing effect of fibers on asphalt. A high asphalt adsorption rate is conducive to preventing asphalt mastic segregation and asphalt overflow under high temperature conditions and better high-temperature performance of asphalt.

The leakage test revealed that 6 mm BFs had the lowest asphalt mastic mass loss rate, followed by BFs with a length of 9 mm and 15 mm [15]. This suggests that 6 mm BFs have a greater capacity for asphalt adsorption than the other two fiber lengths. The improved impact of the three BF lengths is mainly determined by their specific surface area and contact area with the asphalt. Lignin fiber offers superior asphalt adsorption characteristics compared to BF and polyester fiber. Since lignin fiber is made from natural wood, its surface is the roughest of all fibers. Due to its highly flat surface, synthetic polyester fiber has the worst asphalt adsorption characteristics of the three fibers [15]. Using a JJYMX-1 asphalt binder adsorption tester, the findings showed that the lignin fiber adsorption rate was 70%, the BF adsorption rate was 62%, and the polyester fiber adsorption rate was 34%, which may be due to the high number of voids in the lignin fibers [52]. Liu et al. [35] suggested the ratio of asphalt adsorption by BFs to fiber mass for evaluating the asphalt adsorption capability of BFs, and the proposed silane-coupling agent improves basalt and asphalt compatibility by increasing BF meshing chemical bonding. Section 5 contains a detailed description of studies on BF modifiers.

## 3. The Preparation Process of the Basalt Fiber Asphalt Mixture

The dispersion uniformity of fibers, as a non-directional reinforcement of asphalt mixture, has a significant impact on the enhancing effect. It is usually considered that the fibers in the mix should be avoided into a cluster, as far as uniform distribution is feasible [53]. Table 2 lists the current operating approaches researchers employ in the investigation of BF asphalt blends. There are currently three basic mixing procedures for BF asphalt mixes: wet mix, dry mix, and a combination of the two [54]. The wet mix method uses a high shear mixer, and fibers are first combined with binder before being mixed with aggregates. In the dry mix method, fibers are added to aggregates before being mixed with the binder. According to prior studies, the dry mix approach is superior to the wet mix method since it does not require a high shear mixer.

The final approach is a combination of dry mix and wet mix methods; aggregates and binder are combined first, and then fibers are added. This method results in a mixture with fewer fiber agglomerations and better fiber dispersion. Furthermore, the disadvantage of fiber agglomeration with the dry mix method is smaller than that of the wet mix method [54]. Future studies could develop the dispersion process of commercial BF before mixing or a better preparation method for the mixture. Meanwhile, it is also necessary to create a new strategy to characterize the dispersion of the fiber in the mixture, which is of great significance in studying the fiber strengthening mechanism.

## 4. Performance of Asphalt Mixture Modified with Basalt Fiber

### 4.1. High Temperature Performance

The addition of BFs can significantly reduce rutting depth and increase the dynamic stability of asphalt mixes. When the number of loadings reached 20,000, the added fibers content was equal to 0.3% by weight of aggregate rutting depth and was reduced by about 30% by the wheel tracking test [21]. Zhang et al. [26] observed similar phenomena in the Hamburg rutting test of OGFC mixes, where fibers can effectively reduce the rutting depth, and proposed the optimal BF dosing of 0.15%. In comparison to unmodified asphalt mixture, the dynamic stability of asphalt mixes containing 0.4% BFs was raised by 40%, according to Ye et al. [59]. Zhao et al. [20] added 0.3% BF to the asphalt mixture, and dynamic stability reached the maximum value of 5568 times/mm, 1.25 times the dynamic stability value of the asphalt mixture without BF. The light components in asphalt may be adsorbed by BF, increasing the viscosity of the asphalt mortar. Furthermore, the fiber bridging effect increased bonding strength. However, Guo et al. [52] found that the dynamic stability of the mixture first increased and then decreased with the BF content, reaching a peak at 0.4% fiber content. When the fiber content exceeds the optimal value, the fiber agglomeration resulting from uneven fiber dispersion in the asphalt mixture and the excessive asphalt adsorption will reduce the high temperature stability.

Thanh et al. [29] compared other commonly used fibers for asphalt mixture reinforcement and found that the dynamic stability of SMA decreased rapidly when the temperature increased, in which BF SMA decreased the slowest. The decrease in dynamic stability was 57.7%, 47.4%, and 57.5% for lignin, basalt, and polyester fiber SMA, respectively. The reason for this is the high tensile strength of BF, which has better resistance to high temperature than polyester fiber and lignin fiber. As stated in Section 2.1.2, the physical and mechanical properties of BF did not change at high temperatures, so it had little effect on the performance of the blends. Similarly, the study by Wang et al. [22] reported that the dynamic stability of the BF modified asphalt mix (0.34% admixture, 6 mm length, 6.57% asphalt aggregate ratio) was improved by about 25.3% compared to the lignin fiber modified asphalt mix (0.4% admixture, 1.1 mm length, 6.8% asphalt aggregate ratio) in the best fit case.

Huang et al. [33] further studied the performance of SMA; it was studied with a total fiber blend of 0.4% and five ratios of 0:4, 1:3, 2:2, 3:1, and 4:0 between lignocellulosic and BFs. In the rutting tests, the dynamic stability of the mixture steadily enhanced with increasing the BF ratio, indicating that the BF improved the rutting resistance performance more significantly. The incorporation of BF in SMA creates a strong network of fibers that restricts the mobility of the mastic, strengthening the deformation resistance of the asphalt mixture. Another reason why other fiber combinations did not improve dynamic stability significantly is that when fiber is added, the asphalt content increases at the same time, which increases permanent deformation at high temperatures. Fibers can improve the permanent deformation resistance of asphalt mixture, although the impact is minor. Lou et al. [60] studied the effect of different fiber lengths and mix grades on dynamic stability. For blending with 0.3% BF, fiber lengths of 3, 6, 9, 12, 15 mm and mix types of SUP-13, SUP-20, SUP-25 were used. Compared with the asphalt mixture without BFs, the dynamic stability increased by 12.2–29.9%, and shear strength increased by 19.1–29.6%. The best reinforcement effect was found for SUP-13 at a fiber length of 6 mm, SUP-20 at fiber lengths of 6 and 9 mm, and SUP-25 at 9 and 12 mm fiber lengths. The variation in fiber length has a minor impact on the high temperature deformation resistance of HMA.

In addition, Lou et al. [61] reported the high-temperature deformation performance of AC-10 and SMA-10 blended with different contents and lengths of BFs. The data showed that for AC-10, when 3 mm BFs with 0.2%, 0.3%, 0.4%, and 0.5% content were added, the dynamic stability of the ultra-thin abrasion layer was improved by 99.3%, 69.7%, 31.0%, and 21.8%, respectively, while the rutting depth percentages were reduced by 31.7%, 23.0%, 18.3%, and 8.9%, respectively, compared with pure asphalt. For one thing, BFs can increase the viscosity of asphalt, and for another, the network structure formed by BFs can restrict the relative movement of aggregates. The higher fiber content tends to agglomerate the excess fibers during mixing, affecting fiber dispersion and the performance of the ultra-thin abrasive layer. Similarly, the best high-temperature resistance to deformation of the ultra-thin abrasive layer was achieved at 0.2% of 6 mm BFs. Overall, under the same doping condition, 0.2% doping is the best, and the reinforcement effect of 3 mm BF is better than 6 mm. For SMA-10, the reinforcement effect is the best when the fiber content is 0.3%. Under the same doping condition, 6 mm BF is better than 3 mm, inconsistent with the AC-10 grade. Since SMA grade is a skeleton structure with a larger spacing of coarse aggregates, longer fibers can play a better connecting role. The optimal percentage of basalt fiber is 0.15–0.34%, with a fiber length of 6 mm–9 mm. Rutting depth can be reduced by around 30%, and dynamic stability can be enhanced by roughly 25–40%.

### 4.2. Low Temperature Performance

The inclusion of BFs in asphalt mixes can improve their low-temperature crack resistance. The maximum bending strain of the asphalt mixture may be enhanced by 24.8% by adding 0.4% BF. BFs are randomly distributed in the three-dimensional space of the asphalt mixture, allowing stress to be transferred and distributed while avoiding excessive stress concentration [20]. Wu et al. [46] conducted the three-point bending fracture test and showed that the modulus of stiffness of the asphalt mixture with 0.3% BFs increased by 2.5–39.2% compared to the asphalt mixture without fibers. Meanwhile, the variation of stiffness modulus of asphalt mixture doped with lignite fibers was unstable, and their low temperature crack resistance was unstable.

Similar to the effect of BF on the improvement of high temperature performance, there is also an optimal amount of doping on the improvement of low temperature effect. Li et al. [39] conducted a three-point bending beam test and found that 0.4% was the optimum doping for AC-13 at −20 °C to increase the bending stress. This is due to the BFs and the mixture forming a composite structure that can sustain external loads. When the number of fibers exceeds the optimum, the fibers accumulate, thus reducing the bending stress. Similarly, the AC-20 also had a similar “increase-decrease” tendency, and the optimum fiber content is 0.3%. Guo et al. [52] came to similar conclusions; the maximum tensile stress and strain increased with the fiber dose and then decreased. The maximum tensile stress and strain increased by 24–25% at 0.4–0.5%.

Lou et al. [61] further investigated the effect of mix grading and fiber length on low-temperature properties by low-temperature bending beam tests. For AC-10, the maximum breaking strain and the minimum bending stiffness modulus were found when 0.2% of 3 mm BFs were added. For SMA-10, the fracture strain was maximum when the mass content of 6 mm BF was 0.3%. This is due to the fact that SMA-10 has a skeletal structure. Since space between the aggregates is larger than that of AC-10, it gives SMA-10 a greater breaking strain at a fiber length of 6 mm. BFs act as a “bridge” to effectively prevent the crack initiation and expansion in the asphalt mixture, increasing breaking strain and decreasing flexural stiffness modulus. The variation in fiber length also significantly affected low-temperature crack resistance, probably because the fiber length affected the embedding depth at the fracture of the mixture [60].

Huang et al. [33] have studied the effect of mixed fibers (cellulose fiber and BF) on the performance enhancement of the asphalt mixture. It is found that, with the increased proportion of BF, asphalt mixture deflection strength and stiffness modulus increase while maximum tensile strain decreases. Conversely, when more cellulose is added, asphalt mixture deflection strength and stiffness modulus decrease while maximum tensile strain increases. This results from different mechanisms of the two fibers. BF is a tough material with a large modulus. BFs form a network that helps to transmit internal stresses, improving the stiffness of SMA. The primary impact of cellulose is to raise the asphalt content, which is thought to improve sample ductility. The addition of cellulose and BF appears to boost both the deflection strength and ductility. The maximum tensile strain increased by 5.3% when cellulose: basalt = 3:1, and the deflection strength increased by 7.1% compared to the asphalt mixture without fibers.

According to the direct tension test (DTT), the fiber content added to the asphalt should not exceed 1.3% of the weight of the asphalt. According to the fatigue test, fiber incorporation can reduce the stress concentration between the fillers and eliminate fatigue damage in the interface region [25]. Based on the indirect tensile stiffness modulus (ITSM) test, the stiffness modulus increased by 22.5% and 38.2% at 5 °C and 20 °C when 0.4% fiber was added [32]. Another study showed that the indirect tensile stiffness modulus of the BF modified asphalt mix (0.34% admixture, length 6 mm, asphalt aggregate ratio 6.57%) was 50.6% higher than that of the lignin fiber modified asphalt mix (0.4% admixture, length 1.1 mm, asphalt aggregate ratio 6.8%) in the best fit case [22]. The results of the various test techniques all show that BF improves low temperature performance of asphalt mixture.

Guo et al. [37] used the digital image correlation (DIC) technique to measure the changes in the fracture resistance of glass fiber, steel fiber, and BF asphalt mixtures in the IDT test in real-time. After fiber reinforcement, it was seen that the fracture route of the asphalt mixture curved more, proving that the fibers improved the tensile direction of the asphalt mixture. In the plastic phase, the duration of the control mix was about 10.5 s, while the time of the glass fiber, BF, and steel fiber mixes were 13.7 s, 19.7 s, and 8.7 s, respectively. It can be concluded that BFs are the best choice for mixture modification, and the 6 mm fibers exhibit better fracture retarding efficiency than the 12 mm and 20 mm fibers. Jiao et al. [58] used the acoustic emission (AE) technique to evaluate the damage fracture performance of BFs on AC-13 asphalt concrete under low-temperature indirect tension test (IDT) conditions. The results showed that incorporation of 0.5% length of 6 mm and 12 mm BFs at −10 °C IDT conditions could improve 38.8% and 57.0% failure load. The advantage of 12 mm BFs in improving the ductility of asphalt mixes was more obvious. Fu et al. [28] utilized the acoustic emission technique to investigate the failure load of BF asphalt mixes splitting at −10 °C. The addition of BFs increased the breaking load of the mix by 38.8%. Tensile cracking was the dominant crack pattern under the Indirect tensile test in the control asphalt mix and the BF incorporated asphalt mix. The percentage of tensile cracking was reduced by 8% with fibers to the asphalt mixture.

In some cases, the BFs did not improve the low temperature performance of the mixes as intended. Polypropylene fiber and hybrid fiber micro-surfacing, for example, outperform BF micro-surfacing in terms of low temperature cracking resistance. The reason for this is that polypropylene fiber has a higher breaking elongation than BF. The higher the breaking elongation of fiber, the greater its toughness. As a result, it is also difficult to draggle the fiber [30]. Fu et al. [49] based on the Burgers model found that the addition of 6% BFs adversely affected the low temperature creep performance of asphalt mixes. The addition of nano-TiO_2_/ZnO may be able to reduce the negative effects and improve the low-temperature performance. Davar et al. [48] found that compared with the control asphalt mix, the tensile strength of the mix with 0.3% BFs increased by 4.6% in the −5 °C low temperature indirect tensile strength test, and the tensile strength of the mix with 0.3% BFs increased by 22% when diatomaceous earth powder was added. Therefore, the low temperature performance of asphalt mixes can be further improved by the addition of diatomaceous earth powder.

### 4.3. Water Resistance Performance

The inclusion of BFs in asphalt mixes can improve their water resistance. Cetin et al. [45] found that the indirect tensile strength value at 0.6% of BFs was 75.6% higher than that of the control group. According to the quantitative results of the logistic damage model, for 0.4% dosing under 6 mm BF makes AC-13 mix, compared with the control group, the degree of freeze-thaw damage of asphalt mix is significantly reduced by about 25%, and the damage growth rate is significantly decreased by about 45% [62]. Cheng et al. [32] conducted freeze–thaw cycle tests on BF asphalt mixtures and found that the addition of 0.4% BF increased the splitting strength by 23%–51% and the indirect tensile stiffness modulus by 35%–75% over 15 freeze-thaw cycles. This is due to the adsorption action of the asphalt and the BF. Exfoliation of the aggregates under water action was difficult due to the higher adhesion capacity between the asphalt and the aggregates. BFs formed a spatial networking structure in the asphalt mixture, acting as reinforcement and toughening. Lou et al. [61] for the effect of fiber admixture and length concluded that for AC-10, the residual Marshall stability values were greatest when the 3 mm BF content was 0.2% or when the 6 mm BF content was 0.3%. In SMA-10, the water stability index is higher than other mixes with the addition of 0.3% BF (3 mm or 6 mm). This is because excessive fiber content can easily lead to curling and entanglement, which may cause an area in the mix where the fibers collect with each other, leading to stress concentration upon failure. BFs can act as a bridge, binding together the asphalt inside the mix, a phenomenon that contributes to water resistance.

In the water immersion Marshall test, the residual stability first increased and then decreased, and the peak value appeared when the BF content was 0.4%, and the residual stability increased by 9.1%. When the fiber content is less than 0.4%, the interfacial adhesion between fiber and asphalt is improved, and the water resistance is improved. When the dosage of asphalt binder exceeds the optimal dose, the skeletal structure of the mixture is destroyed and the mechanical strength under wet circumstances is reduced. In the freeze-thaw splitting test, the tensile strength ratio (TSR) peaked at 89.9% with a BF content of 0.4%, which improved by 13.5% [52]. In the study of Zhao et al. [20], the optimal amount of BF for water sensitivity was 0.4%, when the TSR value could be improved by 8.3%. The value of TSR is dramatically diminished when the BF dose exceeds 0.4%. BF adsorbs light components in asphalt, thickens the asphalt layer, and enhances the bonding strength between the aggregate and the asphalt, which helps to prevent moisture from accessing the asphalt-aggregate interface. Excessive fiber dosage, on the other hand, causes uneven fiber dispersion and excessive porosity of the mixture, reducing the water resistance.

To study the effect of different fiber types on asphalt mixes, Kong et al. [41] conducted a dry and wet splitting test at 15 °C. The test results show that the mixture with a BF content of 0.3% has a better anti-splitting performance. The splitting resistance of BF is better than that of polyester fiber (PET). The residual Marshall stability and tensile strength of the BF modified asphalt mix (6 mm, 0.34%, asphalt aggregate ratio 6.57%) increased by 9.3% and 5.2% compared to the lignin fiber asphalt mix (1.1 mm,0.4%, asphalt aggregate ratio 6.8%) at the optimum mix ratio [22]. The TSR of BF and cellulose SMA were 90.8% and 89.0%. The best moisture stability was achieved when the ratio of cellulose fiber to BF reached 2:2, with a TSR of 93.2%. This is due to the compound effect of asphalt adsorption by cellulose and strength enhancement by BFs, improving its moisture resistance [33]. To simulate the water resistance of fiber-reinforced asphalt mixes in saline and harsh humid environments, Zhang et al. [63] found that the residual stability of asphalt mixture with BF, polyester fiber, and polyacrylonitrile fiber increased by 9.4%, 7.2%, and 6.1%, respectively, while the freeze–thaw splitting strength ratios increased by 21.6%, 19.9%, and 15.4%, respectively, in comparison to the control group at the optimal dosing. In humid and salty environments, BF can also improve the water resistance of asphalt mixes.

### 4.4. Mechanical Property of Asphalt Mixture Modified with Basalt Fiber

#### 4.4.1. Fatigue Performance of Asphalt Mixtures

BFs can significantly improve the fatigue resistance of asphalt mixes. Radziszewski et al. [44] found that asphalt mixtures with 0.1%, 0.3%, and 0.5% BFs increased the fatigue life by 18%, 48%, and 73% compared to the control group, at an average strain level of 200 μm/m. In addition, Lou et al. [64] reported that the fatigue life N_f,50_ of SMA-13 with BFs increased by 51–185%. The N_f,50_ of SUP-20 with BFs increased by 4–230%, and the N_f,50_ of SUP-25 with BFs increased by 45–362%. The high elastic modulus and superior elongation at the break of BFs can improve the recovery of asphalt deformation and effectively prevent the extension of fatigue fracture. After the addition of BFs, the dissipated energy of the specimens increased. This is mainly because the BFs can withstand part of the stresses, increasing the dissipation energy required to achieve the same strain. Another study reported that 6, 9, and 12 mm were suitable forSUP-13, SUP-20, and SUP-25, respectively. This illustrates that the optimum fiber length is related to the aggregate spacing, influenced by the mix grading [60]. In the four-point bending fatigue test, Wu et al. [56] discovered that the improvement of BF on fatigue life is approximately three times in AC and four times in SMA, indicating that the increase of BF is more substantial in SMA.

For the OGFC mixture, the addition of BFs softened the mixture reduced the stiffness of the mixture continuously with the addition of fibers. The softening phenomenon is mainly due to the introduction of fibers that stabilize the free asphalt. More free asphalt provides more lubrication to the mixture, reducing the mixture’s stiffness. When the optimum amount of BFs is 0.15%, the fatigue life number increases by 188.9% compared to the control group [26].

#### 4.4.2. Toughness of Asphalt Mixtures

In the toughness test, the toughness index (TI) of BFs reinforced asphalt mixture attained a maximum when 0.4% BF. The TI of asphalt mixture modified with 0.3% and 0.4% BF rose by 10.6% and 13.9%, respectively, as compared to AH-70 asphalt mixture. When the BF percentage increased to 0.5%, the TI of BF modified asphalt mixture was reduced by 4.9%. BFs form a three-dimensional network structure that could transfer and disperse stress and reduce crack propagation rate to improve the anti-deformation performance [38].

#### 4.4.3. Rheological Property of Asphalt Mixtures

Zhang et al. [65] obtained the conclusion by compressive creep test; the creep and residual deformation decreased and the creep stiffness modulus increased with the increase of BF content. The bending creep test (BCT) test results showed that 0.1% and 0.2% of fiber reduced the deflection at 3600 s by about 13.1% and 34.6%, respectively, compared with the control specimens. The researchers proposed a three-dimensional numerical model and assumed that the BF-reinforced asphalt-like material consists of a homogeneous asphalt-like matrix (Burgers viscoelastic model) and BFs (Burgers elastic model). 0.1% and 0.2% of BFs decreased shear strain values and compressive strain values for asphalt mastic and asphalt mortar. At constant BF content and diameter, the number of randomly distributed BFs increases as the BF aspect ratio decreases [66]. The best reinforcement is provided by horizontal fiber distribution, which is superior to random fiber distribution [67]. In a further study, by simulating creep deformation in ABAQUS software, fiber content and aspect ratio were the main factors influencing the viscoelastic performance. The viscoelastic deformation results of the SCB model at the bottom of the midspan showed that compared to the AC-13 control, the asphalt mixture with a BF content of 0.3% had a significant reduction in deformation at 1800 s by about 36.1%. The deformation of AC-20 grade was significantly larger than that of AC-13 for both control and BF asphalt mixes, indicating that the asphalt mortar matrix has an important influence on the creep performance of BF asphalt mixes [31]. The effect of fiber length to diameter ratio on flexural–tensile rheological strain was found to be 4.3%, 16.1%, and 32.9% lower for BF with length to diameter ratios of 30, 40, and 50, respectively, compared to BF with length to diameter ratio of 20. As the fiber length to diameter ratio increases, the fibers withstand more stress. However, in practical engineering, the increase in fiber length can lead to agglomeration and make it difficult to achieve the reinforcement effect. Therefore, it is also necessary to consider the construction and control of fiber length [68].

The rheological properties of short-cut BFs (CBF), wool-like BFs, lignin fibers, and polyester fibers integrated with SBS modified asphalt were investigated by Kou et al. [69]. Shape, tensile strength, elastic modulus, and content of fibers are critical elements in defining the rheological characteristics of asphalt mixture. more structural asphalt was adsorbed by flocculent fibers than by bundle fibers. Flocculent fibers have better deformation resistance than bundle fibers.

#### 4.4.4. Cracking Resistance of Asphalt Mixtures

Yang et al. [43] employed the acoustic emission technique to analyze the performance of specimens in semicircular three-point bending tests and concluded that the addition of BFs may block the development of the main macroscopic crack and toughen the mixture. The rapid fracture stage is the main stage during which BFs provide crack resistance and toughening. By using asymmetric semicircular bending tests, Guo et al. [42] found that the addition of BFs reduced the ultimate bearing capacity of the mixture by 14–43% at 20 °C. The bond strength between the asphalt and the fiber decreases with rising temperature, resulting in a decrease in the damage load. Therefore, the bond between BFs and asphalt may be an essential factor affecting the resistance of asphalt to medium-temperature fracture.

The SCB test obtained the maximum fracture toughness value by adding 0.3% BF to the asphalt mixture, and 4 mm BFs were better than 8 and 12 mm [54]. The SCB test findings of the experiment by Wu et al. [56] were that the addition of BF increased the fracture energy of AC-13 and SMA-13 by 36% and 38%, respectively. Pei et al. [70] combined the results of low-temperature beamlet bending test, IDEAL cracking test, and SCB test to conclude that for AC-13, BFs with a diameter of 7 μm had the best reinforcement effect on the crack resistance of the mixture.

Lou et al. [71] analyzed the effect of different aggregate grades on the crack resistance of BF reinforced asphalt mixes and found that the crack expansion rate of SMA-13 mixes was slower than that of SUP-13 mixes. This phenomenon is because SMA has a dense skeletal structure and can disperse stress. In SMA, lignin fibers are commonly used to adsorb the asphalt inside SMA, which helps to strengthen the asphalt inside the asphalt mix. Meanwhile, SUP asphalt mix is a kind of mix with suspended dense structure. Therefore, SMA-13 asphalt mixes have better cracking resistance than SUP-13. For different nominal maximum aggregate sizes (NMAS), the optimum asphalt content of asphalt mixes with larger NMAS is lower, which is not conducive to cracking resistance. Therefore, asphalt mixes with higher NMAS have lower cracking resistance.

Alfalah et al. [40] concluded that 0.15% (BF, fiberglass, and carbon fiber) had no effect on cracking and rutting performance based on the asphalt pavement analyzer and indirect tensile asphalt cracking test (IDEAL-CT). Only carbon fiber, at a dosage rate of 0.3%, enhanced cracking resistance performance without the need for extra binder. This may be due to the different interfaces between fiber and asphalt, which are worth further investigation.

### 4.5. Other Properties of Asphalt Mixture Modified with Basalt Fiber

#### 4.5.1. Skid Resistance

There are fewer reports related to the anti-wear, anti-slip, and aging properties of fiber-reinforced asphalt mixtures, but they are equally worthy of attention. Celauro et al. [21] evaluated the macrotexture of the BF asphalt mixture by the sand patch test, and the pendulum test evaluated the surface slip/skid resistance. Regarding surface texture, the BF asphalt mixture meets the standards for skid resistance, macro-texture, and micro-texture for urban surface courses. When the asphalt content and BFs are increased, the macro-texture decreases. In terms of rutting resistance, the addition of BFs is beneficial. Lou et al. [61] conducted the sand patch test and pendulum test and found that the influence of BF length on slip resistance was not significant. Tanzadeh et al. [47] found that the addition of fibers made the OGFC more abrasive and raised the oxidation sensitivity of the asphalt mixture, and that adding nano-silica was able to reduce the degree of improving the negative impact to some extent. Therefore, further studies are needed for the related pavement application effects of BF.

#### 4.5.2. Long-Term Performances

Long-life pavement has been a hot topic, so the long-term performance study of fiber-reinforced asphalt pavement deserves attention, and obviously, there is less research in this area. By wheel tracking test and uniaxial penetration test, Wu et al. [72] found that the high temperature performance of BF SMA-13 improved with increasing aging, while the aging process of lignin SMA-13 reduced its high-temperature performance. This is because in BF SMA-13, although SBS degradation also leads to a decrease in the high-temperature performance of the asphalt mix, the BFs work better synergistically with the stiffer binder, which ultimately leads to an improvement in the high temperature performance of the asphalt mix. Flocculating lignin fibers can only adsorb excess asphalt, while BFs can cooperate with the asphalt mixture to resist loading. BFs can also improve the low-temperature crack resistance of SMA-13 more than lignin fibers. By SCB test, the fracture toughness of BF SMA-13 was 11.5%, 20.7%, and 32.4% higher than that of lignin SMA-13 under unaged, short-term aged, and long-term aged conditions, respectively. Therefore, it can be concluded that the fracture resistance of BF SMA-13 is higher than that of lignin SMA-13. The residual Marshall stability of BF SMA-13 was 0.74% and 2.76% lower than that of unaged asphalt mixes for the short-term aging treatment and long-term aging treatment, respectively. The corresponding percentage decreases for lignin fiber SMA-13 were 1.42% and 3.18%, respectively. Thus, it is indicated that BFs can improve the strength and water resistance of SMA-13. Other grades of asphalt mixes and the performance changes under different aging conditions are worth further study.

According to Table 3, it is well agreed that the incorporation of BF into asphalt results in higher high temperature performance, low temperature, and better moisture resistance; nevertheless, the effect on the skid resistance is not well agreed upon. At the same time, for the application of BF in non-conventional asphalt binder systems such as cold recycled mixture with asphalt emulsion [73], the lifting effect of BF is not ideal, so for the new binder system or other graded mixes, the further research is needed.

## 5. Surface Modifiers of Basalt Fiber

### 5.1. Interface between Basalt Fiber and Asphalt

For the interface study of BF-reinforced asphalt mixtures, the interface is mainly evaluated quantitatively by parameters such as adhesion and surface energy, and scanning electronic microscopy (SEM) test methods observe the interface. Qin et al. [15] proposed a viscosity-enhancing and stability mechanism, as well as a reinforcement and bridging mechanism. Miao et al. [75] concluded from surface energy tests that the surface energy of asphalt rubber was the largest, and the surface energy of BFs was the largest among four types of fibers (one fiber-reinforced plastic (FRP), two lignin fibers, and one BF) and four types of asphalt (90#, 70#, asphalt rubber, and styrene-butadiene-styrene (SBS) modified asphalt). Given that the higher the surface energy of the fiber, the better the reinforcement effect of the fiber, additional studies to strengthen the interfacial bond between asphalt and fiber is warranted.

### 5.2. Surface Modifiers of Basalt Fiber

The modifiers utilized to enhance the performance of the BFs and asphalt contact are summarized in Table 4. Xiang et al. [76] modified BF with a silane coupling agent and found that the composite modulus of the modified BF asphalt mastic increased, because a more robust structural layer was formed between the modified fiber and the asphalt, possessing stronger mechanical properties, which could improve the stress profile and internal stress distribution of the fiber mastic. The recommended dose of modified fibers is 0.3–0.6%. The rutting coefficient of modified fiber asphalt mastic is higher than that of unmodified because the modified fibers play the multi-directional bridging reinforcement. The modified fibers also enhance thermal stability, reducing shear deformation at high temperatures more effectively. The modified fibers were rougher, and their surface area increased. A silane coupling agent film and protrusions of different shapes and thicknesses were accumulated, which led to a significant development of multi-directional bridging reinforcement of the modified fibers. A firmer structural layer is formed between the modified fibers and the asphalt, which improves the adsorption and stabilization capacity of the modified fibers to the asphalt. It can be concluded that the silane coupling agent modified fibers enhance the rheological properties, including flexibility, shear deformation resistance, crack resistance, and the ability to disperse stress. Following treatment with a silane coupling agent, the surface apparent contact angle of fiber rose from 63.3° to 87°, and the hydrophobicity of fiber increased. The free silane groups in BF treated with silane coupling agent interacted with asphalt, resulting in improved fiber-asphalt interfacial contact. There is significantly greater contact between fibers and asphalt matrix during the pull-out process of modified fiber. Modified fiber reinforced asphalt has much greater deformation resistance than fiber asphalt [36].

Since BF is generated by rapid drawing after high temperature melting and has a smooth surface, it has the drawback of low oil adsorption and easy segregation in road engineering applications. Liu et al. [35] precoated the surface of BF with silane coupling agent (ammonia propyl triethoxysilane, or KH550). KH550 solution improved the surface properties of BF, formed stable Si-O bonds, enhanced the cohesion between BF and asphalt, and the modified BF had heat resistance. Compared with the original BF, its asphalt adsorption capacity increases by 40.3%, and the segregation and dispersion properties greatly improve, increasing the thickness of asphalt film. The experimental results of Lou et al. [77] showed that the work of adhesion between BF and SBS modified asphalt was 42.93 mJ/m^2^, whereas the work of adhesion between modified BF and SBS modified asphalt was 51.64 mJ/m^2^. The formation of a significant number of amino (-NH_2_) groups on the surface of BFs by silane coupling agent is thought to be helpful to the bonding of BFs with asphalt, thus increasing the bonding capabilities of both. The fiber treated by KH-550 improves asphalt surface wetting, resulting in better interfacial adhesion. The 2.0% solution concentration and 30 min modification time can make the BF show good interfacial bonding performance, which is beneficial to improve the high temperature performance and low temperature deformation resistance of BF asphalt mastic.

In a further study by Lou et al. [9], polyvinyl acetate emulsion (PAE), polyester emulsion (PE), and polyol ester emulsion (PEE) were proposed as BF modifiers. Polyvinyl acetate emulsion-modified fibers contributed the most to the high temperature deformability of the asphalt mixes. In the low-temperature flexural beam test, polyvinyl acetate emulsion modified fiber mixes had the lowest modulus and the highest breaking strain. It indicates that polyvinyl acetate emulsion modified fibers may have better interfacial bonding properties with asphalt. Although BFs with different types of sizing agents significantly improved the high temperature deformation resistance and low temperature cracking resistance of asphalt mixes and significantly increased their fatigue life by 500–800%, the impact on water resistance needs additional investigation. As the preferred polyvinyl acetate emulsion. Wu et al. [55] found that the pH values of BFs coated with polyvinyl acetate emulsion, polyester emulsion, polyalcohol ester emulsion, unmodified BFs, and lignin fibers were 7.1, 6.6, 7.1, 7.4, and 7.6. Meanwhile, it was observed by microscopic images that the fiber-asphalt bonding interface was more desirable in the asphalt of basalt and lignin fibers coated with polyvinyl acetate emulsion. More study is needed to determine the best modifier to use based on the overall performance improvement of the surface modifier on the asphalt mixture.

## 6. Conclusions and Recommendation

This article reviews the past literature relevant to the studies on the basalt fiber reinforced asphalt mixture for pavement applications, including its advantages and limitations. Some main findings and suggestions are listed below based on the discussion and analyses of current literature works:

1. Basalt fiber has great mechanical and physical properties and is a green and environmentally friendly fiber with promising applications. By dry mix method (aggregate and fiber mixed first) and combined mix method (aggregate and asphalt mixed first), preparation can be obtained with good dispersion. Basalt fibers form a three-dimensional network structure in the asphalt, which can disperse stress and delay cracks development. Excessive fibers lead to agglomeration in the asphalt mixture, which reduces performance.
2. Basalt fiber with a dosage of 0.15–0.34% and a length of 6–9 mm considerably enhances the high temperature performance of the asphalt mixture. Basalt fiber can reduce the rutting depth in asphalt mixture by about 30% and improve the dynamic stability by 25–40%. Basalt fiber doping in 0.3–0.5%, length of 3–12 mm considerably enhances the low temperature performance of the asphalt mixture. To get the best water resistance performance, basalt fibers are recommended at 0.2–0.4%.
3. After surface modification, the adhesion between basalt fibers and asphalt increases, which can better play the role of a Viscosity-enhancing and stability mechanism and a Reinforcement and bridging mechanism. Polyvinyl acetate emulsion and silane coupling agents are suitable surface modifiers for basalt fibers.

The enhancement effect of BF is influenced by the asphalt type and mix grade, so the selection of specific fiber parameters needs to be studied for applicability. One of the aspects to consider in the development of surface modifiers is the composition of the raw basalt ore, which impacts the binding sites on the surface of the BFs. The surface modifier modifies the surface attributes of the basalt fiber surface, such as hydrophilicity and acidity, affecting the binding between the fiber and the bitumen. Further research is recommended on the long-term life performance studies and life cycle assessment of basalt fiber reinforced asphalt mixtures.

## Figures and Tables

**Table 1:** Basic properties of basalt fiber in asphalt mixtures.

Length (mm)	Diameter (µm)	Density (g/cm^3^)	Relative Density (g/cm^3^)	Heat Resistance (°C)	Melting Point (°C)	Moisture Content	Tensile Strength (MPa)	Elastic Modulus (GPa)	Fracture Elongation (%)	Specification	Ref.
1.5	11–13	-	2.416	-	1500–1600	-	>2000	-	-	-	[20]
3–5	-	-	-	-	1350	-	-	84	2.8	ASTM D2256	[21]
3, 6, 9	13	-	-	-	-	-	≥3000	-	3.2	-	[22]
3, 6, 9, 12	13	-	2.71	-	1600	-	2218	-	-	-	[23]
3, 9, 12, 15	16	-	-	-	-	-	2200–2500	-	2.71	-	[24]
4.5	13	-	-	-	-	-	4100–4840	93.1–110	3.1–3.2	-	[25]
6	9–12	2.63	-	-	1450	-	3800–4840	-	3.1	-	[26]
6	13	2.817	-	-	1500	-	2000	-	-	-	[27]
6	13	2.7	-	-	-	-	3000–3500	-	2.7	-	[28]
6	15	2.463	-	-	-	-	3000–4840	-	-	-	[29]
6	21	-	-	-	-	-	≥1500	93.1–110	3.2	-	[30]
6	20	2.7	-	-	-	-	4500	100	3.1	-	[31]
6	13	-	-	-	-	-	3200	>40	3.2	-	[32]
6	13	2.56–3.05	-	-	-	-	4100–4830	90–110	3.0–3.3	ASTM D 3800-16, ASTM D 5034-17	[33]
6	8	2.5	-	-	1505	-	3000	-	3	-	[34]
6, 9, 15	17	-	-	-	1050	<0.2%	4000–4850	90	>3.2	ASTM D 276-00a, ASTM D 2987-88, ASTM D 5034-95, ASTM D2130-90, ASTM D 204-02	[15]
6–12	12–15	-	-	-	1450–1500	<0.1	2800–3800	90–110	3.2	-	[35]
6–12	12–15	2.56–3.05	-	180	-	-	2800–3800	90–100	3.2	-	[36]
6, 12, 20	13	-	-	-	-	-	3200	-	2.5–2.8	-	[37]
9	16	-	2.71–2.65	-	-	-	2000–2500	>80	-	-	[9]
9	17	2.64	-	-	-	-	3600	90	3.9	-	[38]
9	13–15	-	-	-	1050	-	3000	105	3.1	-	[39]
9	-	-	-	-	2500	-	2500	-	-	ASTM D3800, ASTM D276, ASTM D7138, ASTM D2256, ASTM D204, ASTM D5103	[40]
10–12	12–14	-	2.4	170	-	-	2800–3400	80–90	3.1	-	[41]
12	13	-	-	-	-	-	3200	78.6–94.2	2.5–2.8	-	[42]
12	13	-	-	-	-	-	2700	65	2.5–2.8	-	[43]
12	13–20	2.8	-	-	1450	-	4840	-	3.15	-	[44]
12	9–23	2.6–2.8	-	-	1450	-	4840	89	3.2	-	[45]
12	15	1.36–1.4	-	-	1050	-	3900	-	3.2	-	[46]
24	18	-	-	-	-	-	1100	89	3.15	RBR 18-T 10/24	[47]
24	18	-	-	-	-	-	4100–4850	89	3.1	-	[48]
-	-	2.8–3.3	-	-	-	-	3000–4500	91–110	3.2	-	[49]

**Table 2:** The preparation process of the basalt fiber asphalt mixture.

Mixture Type	Binder Type	Method	Procedures for the Preparation	Ref.
Asphalt binder	SK-90# modified with nanopowder	-	1. Nano-modified asphalt kept at 160 °C. 2. BF was added while stirred. 3. The asphalt was sheared twice for 40 min at 2500 rpm.	[49]
Asphalt matrix	70#	-	1. Asphalt was heated for 1 h at 145 °C. 2. BF mixed with asphalt for 20 min at 145 °C.	[36]
Asphalt mastic	SBS modified asphalt	Dry mix	1. BF and mineral powder were dried at 120 °C. Asphalt heated to 175 °C. 2. BF and mineral powder were mixed. 3. BF and mineral powder mixture were added to the asphalt in 3 portions, at 1000 r/min for 30 min. At 500 rpm for 20 min.	[55]
(AC-16) enhance with diatomite powder	PG 64-22	Dry mix	1. Asphalt and diatomite were heated to 135 °C for 4 h, then mixed for 15 min. 2. BFs and heated aggregates were mixed. 3. Asphalt was added and mixed for 5–6 min.	[48]
(OGFC-10)	PG 82-22 polymer modified asphalt	Dry mix	1. BF was mixed with pre-heated aggregate for 1 min. 2. Asphalt was added and blended for 2 min at 180 °C.	[26]
SMA-13	SBS modified asphalt	Dry mix	1. The aggregates and fillers were baked at 180°C for 2 h. SBS-modified asphalt heated to 170 °C. 2. The aggregates and BF were mixed. 3. Asphalt was mixed with aggregates at 165 °C. 4. The fillers were added and mixed at 165 °C.	[22]
AC-13	SBS modified asphalt	Dry mix	1. BF is mixed with aggregate for 90 s. 2. The asphalt and mineral filler were added and blended at 75 rpm for 180 s.	[20]
AC-13	AH-90	Dry mix	1. The pre-heated aggregates mixed with BFs, for 90 s. 2. The pre-heated asphalt was added and blended for 90 s. 3. The limestone powder was added and blended for 90 s.	[32]
SMA-13, AC-13	SBS modified asphalt	Dry mix	1. BFs were mixed with the aggregate for 90 s. 2. The asphalt was added and blended.	[56]
(SMA-16)	B50/70, SBS modified asphalt	Dry mix	1. Aggregate and BF were mixed for 2 min and then heated at 170 °C for 2 h. 2. Asphalt was heated to 145 °C and then mixed with the aggregate for 2 min.	[45]
AC-13	70#	Combined mix	1. Aggregate and asphalt were blended at 160 °C for 90 s. 2. BF was added and blended for 90 s. 3. Mineral filler was added.	[43]
(SUP-13)	PG 76- 22	Combined mix	1. Heated aggregates and asphalt were mixed. 2. BF is added to the mix in four batches, 15 s apart.	[40]
(OGFC-16)	70# modified with nano-silica	Combined mix	1. Nano-silica and SBS mixed with 1800 rpm at 160 °C, for 20 min. 2. Aggregates and lime powder were mixed with asphalt. Fibers were added.	[47,57]
(AC-13)	AH-70	Combined mix	1. Pre-heated aggregates were mixed with asphalt for 90 s. 2. BFs were added and mixed for 90 s. 3. The mineral filler was added.	[37]
AC-16	A-70	Combined mix	1. BF and mineral filler were dried at 60 °C for 1 h. The asphalt was heated at 160 °C. 2. Asphalt and mineral filler were mixed for 3 min. 3. BFs were added and mixed at 2000 rpm for 5 min.	[15]
(AC-13)	AH-70#	Combined mix	1. Aggregates and asphalt were blended for 90 s at 170 °C. 2. BF was added and mixed for 90 s. 3. The mineral filler was added.	[42]
AC-13	AH-70#	Combined mix	1. Asphalt and aggregates were mixed for 90 s. 2. BFs were added and mixed for 90 s. 3. Mineral filler was added and mixed for 90 s.	[58]

**Table 3:** A summary of research on basalt fiber for asphalt.

Gradation of Asphalt Mixture	Fiber Length	Fiber Dosage	Major Findings	Country, References
AC-10, SMA-10	3, 6 mm	0.2, 0.3, 0.4, 0.5% by weight of mixture	Improved comprehensive performance of the ultra-thin wearing course. 3 mm is suitable for AC-10, 6 mm is suitable for SMA-10. 0.2% fiber content is optimum for AC-10, 0.3% fiber content is optimum for SMA-10.	China [61]
AC-13, AC-20	9 mm	0.2, 0.3, 0.4, 0.5% by weight of mixture	The bending stress, bending strain, and strain energy density first rise and then decrease with increasing BF content. Improved resistance to low temperatures. For low-temperature performance, the optimum fiber content is 0.4% for AC-13 and 0.3% for AC-20. Increased bending strain and strain energy density. Improves the integrity, disperses stress, and slows microcrack extension.	China [39]
AC-13	6 mm	0.3% by weight of asphalt concrete	Decreased flexural-tensile strain. As the fiber distribution shifts from vertical to horizontal, the reinforcing effect steadily rises. Random fiber distribution has a stronger reinforcing impact than vertical and 45° oblique fiber distributions, although it is weaker than horizontal fiber distribution.	China [67]
(OGFC-16) modified with nano-silica	24 mm	0.2% by weight of asphalt	Caused an increase in abrasion. Glass fiber outperformed BF in terms of increasing tensile strength. Glass fibers outperform BF in terms of water resistance, and performance may be enhanced by the addition of nano-silica.	Iran [47]
(OGFC-10)	6 mm	0.15, 0.3, 0.45% by mixture mass	0.15% optimum fiber content. BF exhibits considerable tensile strength and enhances the mixture’s mechanical properties.	China [26]
(AC-20)	no mentioned	0.25, 0.5, 0.75, 1, 1.5, 2% by the weight of mixture	Enhanced Marshall stability. 5% asphalt content and 0.5% BF addition is optimal.	Turkey [74]
AC-13	9 mm	0.2, 0.3, 0.4, 0.5% by the weight of mixture	Based on the strip tensile test, the ultimate tension peaked at 0.4% BF content. Based on the toughness test, the toughness index peaked at 0.4% BF content. The toughness index of the modified asphalt mixture increased by 10.6% and 13.9%, respectively, under 0.3% and 0.4% BF content.	China [38]
(AC-13)	12 mm	0.5% by the weight of mixture	Asymmetric semi-circular bend test: the critical stress intensity factor decreases at medium temperature, but it has minimal influence at low temperature, which increases slightly under mixed-mode conditions. Enhanced the critical fracture energy at medium and low temperatures. Improved the ductility and post-peak bearing capacity. At medium and low temperatures, the generalized maxi-mum tangential stress criterion may be used to predict the fracture starting angle.	China [42]
MS-3 Micro-surfacing	6 mm	0.05, 0.1, 0.2, 0.3% by the weight of mixture	The composite use of polypropylene fibers and BF can improve overall performance and reduce costs. 0.1–0.2% fiber content is optimum. BF has a better dispersion effect than polypropylene fiber.	China [30]
SUP-13, SUP-20, SUP-25	3, 6, 9, 12, 15 mm	0.3% by total mixture weight	BF with varied lengths can further improve the comprehensive performance of HMA to a great extent, especially for the crack resistance related ones. The mass ratio of mixed BF lengths should be 3 mm, 6 mm, and 9 mm = 1:1:1 for optimal Superpave-13 performance. The mass ratio of mixed BF lengths should be 6 mm, 9 mm, and 12 mm = 1:2:2 for optimal Superpave-20 performance. The mass ratio of mixed BF lengths should be 9 mm, 12 mm, and 15 mm = 2:2:1 for optimal Superpave-25 performance.	China [24]
AC-13	6 mm	0.5% by the mass of mixture	Improved fracture resistance. Enhanced the failure load by 38.8%. Reduced the tensile cracking rate by 8%	China [28]
SMA-13	3, 6, 9 mm	1, 2, 2.5, 3, 3.5, 4% by mass of SBS asphalt	Dynamic stability improved by 25.3%. Indirect tensile stiffness modulus enhanced by up to 50.6%. The optimum BF content is 0.34% and the optimum length is 6 mm.	China [22]
SMA-16	6 mm	fiber-aggregate ratio is 0.4%	Improved Marshall stability and dynamic stability.	China [29]
AC13, AC20	6 mm	0.3% by weight of mixture	Enhanced creep resistance and reduced viscoelastic deformation. The effects of fiber content and aspect ratio on the viscoelastic performance of BF asphalt mixture are the main factors. Creep deformation simulations: the influence of BF and aggregate modulus on the creep deformation parameters of the Burgers is insignificant, although increasing BF concentrations and aspect ratios have a positive effect.	China [31]
(AC10)	4, 8, 12 mm	0.1, 0.2, 0.3% by weight of mixture	The fracture toughness of an asphalt mixture decreases as the BF length increases. 4 mm BF is recommended. The 0.3% BF content is recommended to increase the fracture toughness of the asphalt mixture. 0.3% fiber content and a fiber length of 4 mm are recommended.	Iran [54]
AC13	1.5 mm	0.2, 0.3, 0.4, 0.5% by weight of mixture	Enhanced high temperature, low temperature, and water resistance performance. The appropriate dosage of BF is about 0.3%.	China [20]
AC-16	6, 9, 15 mm	0.2, 0.3, 0.4, 0.5, 0.6% by weight of mixture	Enhanced high temperature, low temperature, and water resistance performance. The appropriate dosage of BF is about 0.4%, and the optimum length is 6 mm.	China [52]
AC-13	6 mm	0.2, 0.3, 0.4, 0.5% by weight of mixture	Enhanced high temperature, low temperature, water, and freeze-thaw damage resistance performance. The optimal BF content is 0.4%.	China [32]
Porous asphalt mixture-13	3, 6, 9, 12 mm	0.3% and 0.4% by weight of aggregate	9 mm length and 0.3% BF are suggested. Little effect on water sensitivity.	China [23]
AC-13	12 mm	0.5% by the weight of mixture	BFs had no improvement effect on the bending failure load. The rapid fracture stage is the primary stage in which BFs provide crack resistance and toughening.	China [43]
SMA-13	6 mm	0.4% by the weight of mixture; the ratio of cellulose to BF is 0:4, 1:3, 2:2, 3:1, and 4:0 by weight	Compared to mixes without fiber, the dynamic stability of BF mixes improved by 35%. The simultaneous introduction of cellulose and BFs improves low temperature, fatigue, and moisture damage resistance.	China [33]
asphalt binder	6 mm	1%, 2%, 3%, 4% by the weight of asphalt	Temperature sweep test: The higher flexibility of wool-like BFs, lignin fibers, and polyester fibers reduces the overall stiffness of the asphalt, while the higher stiffness of short-cut BFs improves the fatigue resistance of asphalt mixture. Linear amplitude sweep test: The bundled fiber had a noticeable effect on improving fatigue performance at 1–2%, while the flocculent fiber had a significant impact at 3–4%. The rheological properties of asphalt mixes are affected by the shape, tensile strength, elastic modulus, and content of the fibers. The flocculent fibers adsorbed more structural asphalt than bundle fibers. Flocculent fibers are better suited to improving rutting resistance.	China [69]
AC-13	6 mm; 7, 13, 25 μm	0.3% by the weight of mixture	Improved flexural strength and fracture energy. The crack resistance was improved the most with diameters of 7 μm.	China [70]
SMA-13, SUP-20, SUP-25	6, 9, 12 mm	0.3, 0.4% by the weight of mixture	Increased fatigue life and dissipated energy.	China [64]
SUP-13, SUP-20, SUP-25	3, 6, 9, 12, 15 mm	0.3% by the weight of mixture	6 mm fiber is recommended for SUP-13, 9 mm fiber is recommended for SUP-20, 12 mm fiber is recommended for SUP-25. Improved fatigue life, dynamic stability, and shear strength. The water stability and high temperature deformation resistance are not affected by fiber length. Low temperature bending beam test: the breaking strain increases by 4.7–21.2%, and the flexural modulus is reduced by 1.1–13.6%.	China [60]
AC-11 S	12 mm	0.1, 0.3, 0.5, 0.7% by the weight of mixture	With 0.3% BF, the Marshall stability rose by 7.4%, and the Marshall stiffness reduced by 6.8%. Increased fatigue life.	Poland [44]
SMA-13, AC-13	6 mm	0.1, 0.3, 0.4% by the weight of mixture	BF improves fatigue life by roughly 3 times in AC and 4 times in SMA. The cumulative dissipation energy of BF asphalt mixes increased significantly by 1.8 for AC-13 and about 3 for SMA-13. The fracture energy increases by about 36% for BF modified AC-13 and 38% for BF modified SMA-13. BF SMA-13 seems to have better crack resistance than AC-13.	China [56]
SMA-16	12 mm	0.1, 0.2, 0.4, 0.6, 0.8% by the weight of aggregate	Increased rebound modulus. The best resistance to permanent and high temperature deformation was obtained with 0.4% BF. The ITS values of asphalt mixes with 0.6% BFs were 75.6% and 11.7% higher than control mixes and SBS mixes, respectively. The TSR values of the asphalt mix with 0.6% BFs were 11.0% higher for the control group. The optimal amount of BF is about 0.6%.	Turkey [45]
SMA-13, SUP-13, SUP-20, SUP-25	6,9,12 mm	0.3% by the weight of mixture	IDEAL Test: The Ginitial index increased by 15.4%, 11.9%, 19.6%, 9.3%, and 13.6% for the addition of BFs to the asphalt mix. The crack resistance of BF SMA-13 is higher than that of BF SUP-13.	China [71]
AC-13	15 mm	0.3% by the weight of mixture	Three-point bending fracture test: 2.5–39.2% increase in stiffness modulus of asphalt mixes incorporated with BFs. Improved the crack resistance property and low temperature performance.	China [46]
SMA-13	6 mm	0.4% by the weight of mixture	Long-Term Performance: The high temperature performance of BFs SMA-13 increased with aging, while the aging process of lignin fibers SMA-13 reduced its high temperature performance. Three-point bending fracture test: increased maximum bending tensile strain. SCB test: The crack resistance of SMA-13 with the addition of BFs was better than that of lignin SMA-13. The residual Marshall stability of BFs SMA-13 after short-term and long-term aging was 0.74% and 2.76% lower than that of the unaged treatment, respectively.	China [72]
AC-13	6 mm	0.1, 0.2, 0.3% by the weight of mixture	Reduced flexural-rheological value of the 3D model. The flexural- tensile rheological value of the 3D model decreases with increasing BF aspect ratio. Increasing the fiber length can improve the flexural-tensile rheological properties or reduce rheological strain. The reinforcement effect of BF was better than that of steel wool fiber.	China [68]

**Table 4:** Summary of fiber surface modifiers in basalt fiber asphalt mixes.

Type of Asphalt Mix	Fiber Modified Agent	Agent Concentration	Fiber Dosage	Major Findings	Ref.
Asphalt mucilage	silane coupling agent	1.0%	0.3%, 0.6%, 0.9% by weight of asphaltmucilage	BBR and DSR test: Improved the rheological properties of asphalt mastic. Improved fiber adsorption and stabilization of asphalt. Improved the thermal stability of asphalt mastic.	[76]
Asphalt	silane coupling agent (KH-550)	2.5%	0.5, 1.0, 1.5% by weight of asphalt	The contact angle of the fiber surface increased from 63.3° to 87°, and hydrophobicity increased. Improved fiber-to-asphalt interfacial bonding. Improved resistance to rutting deformation.	[36]
Asphalt	ammonia propyl triethoxysilane (KH-550)	-	5% by weight of asphalt	Formed Stable Si-O bonds, enhanced the cohesive heat resistance of BF and asphalt. Improve asphalt adsorption capacity by 40.3%. Increase the thickness of asphalt film on the fiber surface.	[35]
Asphalt mastic	silane coupling agent (KH-550)	0.5%, 1.0%, 2.0%, and 4.0%	3% by weight of asphalt	Increased surface roughness and surface energy. Improve the maximum pull-out force and work of BF and asphalt mastic. 30 min and solution concentration of 2.0% is optimal.	[77]
AC-20	Polyvinyl acetate emulsion (PAE); Polyester emulsion (PE); Polyalcohol ester emulsion (PEE)	-	0.3% by weight of the mixture	Dynamic stability is improved by 15.75–30.2% at 60 °C and 40.2–81.9% at 70 °C. The rutting depth decreased from 3.9% to 3.1–3.4% at 60 °C and from 7.8% to 6.5–7.2% at 70 °C. The failure strain increased by 12.1–21.6%. The fatigue life extended by 500–800%. Polyvinyl acetate emulsion is optimal.	[9]
Asphalt mastic	Polyvinyl acetate emulsion (PAE); Polyester emulsion (PE); Polyalcohol ester emulsion (PEE)	-	5% by weight of the SBS asphalt	Polyester emulsion makes fiber pH 6.6. Fibers increase the viscosity and modulus of asphalt. BFs modified by PA have an optimal effect on improving high temperature performance	[55]

## Data Availability

Not applicable.
